# Interactive Segmentation of Pancreases in Abdominal Computed Tomography Images and Its Evaluation Based on Segmentation Accuracy and Interaction Costs

**DOI:** 10.1155/2017/5094592

**Published:** 2017-08-31

**Authors:** Hotaka Takizawa, Takenobu Suzuki, Hiroyuki Kudo, Toshiyuki Okada

**Affiliations:** ^1^University of Tsukuba, 1-1-1 Tennodai, Tsukuba, Ibaraki 305-8573, Japan; ^2^JST-ERATO Quantum-Beam Phase Imaging Project, Sendai, Japan

## Abstract

The present paper proposed an interactive segmentation method of pancreases in abdominal computed tomography (CT) images based on the anatomical knowledge of medical doctors and the statistical information of pancreas shapes. This segmentation method consisted of two phases: training and testing. In the training phase, pancreas regions were manually extracted from sample CT images for training, and then a probabilistic atlas (PA) was constructed from the extracted regions. In the testing phase, a medical doctor selected seed voxels for a pancreas and background in a CT image for testing by use of our graphical user interface system. The homography transformation was used to fit the PA to the seeds. The graph cut technique whose data term was weighted by the transformed PA was applied to the test image. The seed selection, the atlas transformation, and the graph cut were executed iteratively. This doctor-in-the-loop segmentation method was applied to actual abdominal CT images of fifteen cases. The experimental results demonstrated that the proposed method was more accurate and effective than the conventional graph cut.

## 1. Introduction

The mortality rate of pancreas cancer has been high worldwide [1]. In order to decrease the rate, pancreas cancers should be detected by medical doctors in their early stages and then treated adequately. Abdominal computed tomography (CT) images have been widely used for the early detection, but it is difficult for medical doctors to diagnose a large amount of CT images in a limited time. Therefore, it is necessary to build a computer-aided diagnosis (CAD) system to assist medical doctors in diagnosing abdominal CT images. The segmentation of pancreas regions in CT images is one of the most important issues in this research field.

There are two types of segmentation method: automatic and interactive. Park et al. proposed an automatic segmentation method of organs in test CT images based on probabilistic atlases (PAs) constructed from CT images for training [2]. Heimann and Meinzer proposed another segmentation method based on statistical shape models (SSM) [3]. These segmentation methods used the PA and the SSM as a priori information of target organs and therefore can work well if the organs in test CT images have similar shapes to those in training CT images. However, if the target organs have different shapes, these methods would fail.

On the other hand, interactive segmentation methods allow medical doctors to deal with irregular organs more flexibly. The medical doctors can give informative clues, such as seed voxels, to CAD systems on the basis of their own anatomical knowledge, and the CAD systems can make use of such information for more accurate segmentation. Barrett and Mortensen proposed a live wire technique to extract target regions by using seed points along the region boundaries [4]. This technique can obtain the shortest path between the seed points but can be applied only to two-dimensional images. Schenk et al. extended the live wire technique so that it can be applied to three-dimensional images [5], but it increased interaction with images. Kass et al. [6] introduced an active contour model to extract target objects. The optimal contour models were obtained by minimizing an energy function to evaluate the fidelity between the objects and the models. The method often produced the suboptimal contour corresponding to the local minimum of the energy function. Sethian proposed the level set method [7], but the method often produced the suboptimal contour, and it is difficult to modify the suboptimal contour intuitively. Grady [8] proposed an interactive segmentation method based on a random walk technique. In this method, several voxels were labeled beforehand, and then the unlabeled voxels were segmented on the basis of probabilities that the unlabeled voxels reach the labeled ones. This method was able to perform well even with ambiguous or discontinuous boundaries, but the segmentation result was still the suboptimal.

The graph cut (GC) technique [9–11] can obtain the globally optimal contours from seed voxels, and many research groups have used the GC technique in their organ segmentation methods. In our previous paper [12], we also proposed a GC-based interactive segmentation method of pancreases in abdominal CT images based on the anatomical knowledge and the statistical information. The anatomical knowledge was represented by seed voxels that were selected by a medical doctor. The statistical information was represented by a PA that was constructed in advance from sample CT images for training. The PA was fitted to the seed voxels through parallel translation, and pancreas regions were extracted by use of the GC technique. The segmentation method was applied to actual abdominal CT images for testing and was evaluated in terms of its segmentation accuracy. In the present paper, we newly adopt the homography transformation that is higher dimensional conversion. The homography transformation can fit the PA to seed voxels of pancreases of more irregular shapes. In addition, we evaluate not only segmentation accuracy but also interaction costs, that is, operation times and efforts, that are newly introduced in this paper and were not evaluated in [12].

This paper is organized by six sections.  Section 2 describes the outline of the proposed method, Section 3 shows experimental results, Section 4 provides a discussion, and Section 5 concludes the paper.

## 2. Outline of the Proposed Method

**Figure 1:**
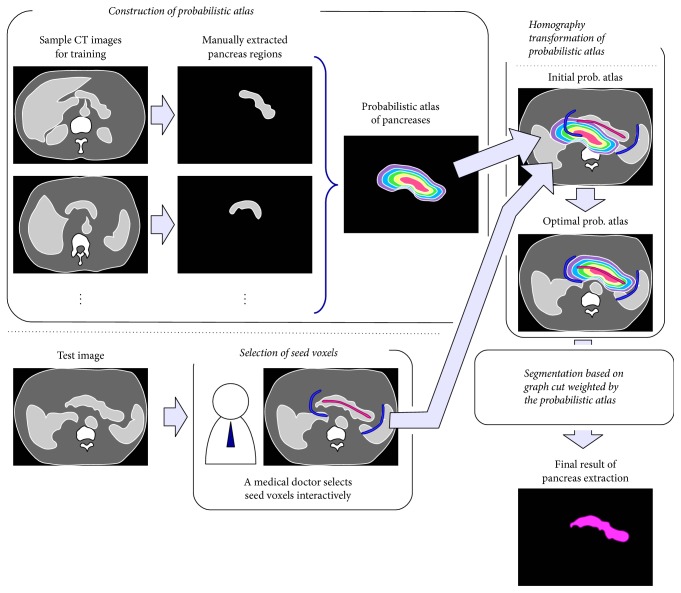
Outline of the proposed method.

Figure 1 shows the outline of the proposed method, which consists of two phases: training and testing. In the training phase, pancreas regions are manually extracted from sample CT images for training, and then a PA is constructed from the extracted regions. In the testing phase, a medical doctor selects seed voxels for a pancreas and background in a CT image for testing by using a graphical user interface (GUI) system. The PA is fitted to the seed voxels by use of the homography transformation. The graph cut technique whose data term is weighted by the transformed PA is applied to the test images. The seed selection, the atlas transformation, and the graph cut are executed iteratively.

### 2.1. Construction of Probabilistic Atlas

A PA is an image each of whose voxels contains the probability of a hypothesis that the voxel is inside a target organ. A probabilistic pancreas atlas is constructed as described below.

First, pancreas regions are manually extracted from sample CT images for training, and boundary boxes are set to the pancreas regions. The CT images are resampled so that the sizes of the boxes are *S*_*x*_ × *S*_*y*_ × *S*_*z*_ voxels. The signed distance transform [13] is applied to the resampled CT images. Positive and negative values are assigned to the voxels of the pancreas and background, respectively. The probabilities of voxels being inside pancreases are calculated by the sigmoid function:

(1) $$\begin{array}{c} \varsigma \left(\;d;a_{p}\;\right)=\frac{1}{1+\exp\left(-a_{p}d\right)}, \end{array}$$

where *d* is a signed distance value and *a*_*p*_ is a gain parameter. Finally, a PA is obtained by averaging the probability values of the corresponding voxels over the resampled images.

### 2.2. Selection of Seed Voxels

**Figure 2:**
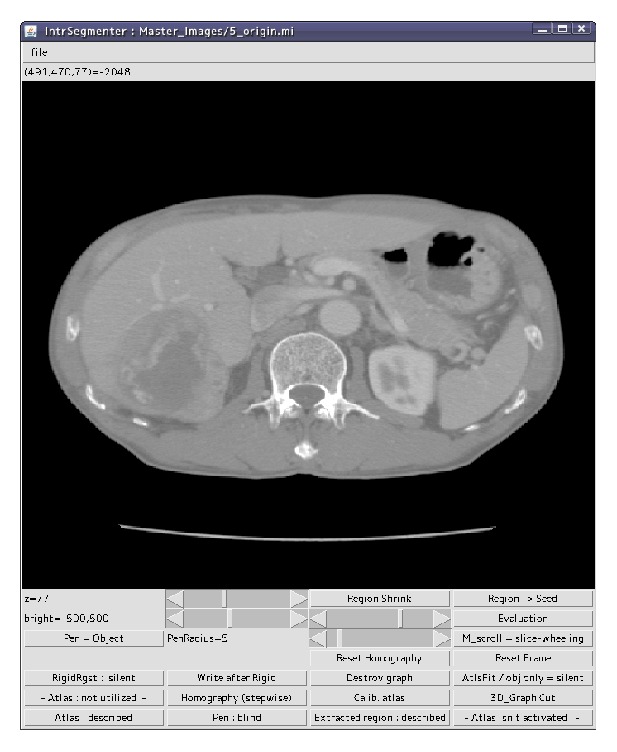
Our GUI system for pancreas segmentation.

A medical doctor selects seed voxels for a pancreas and background in a test CT image on the basis of his or her anatomical knowledge of pancreases. We built a GUI system composed of an image-display area and several buttons as shown in Figure 2. The user can change CT slices with mouse wheel operations and select seed voxels with mouse dragging.

**Figure 3:**
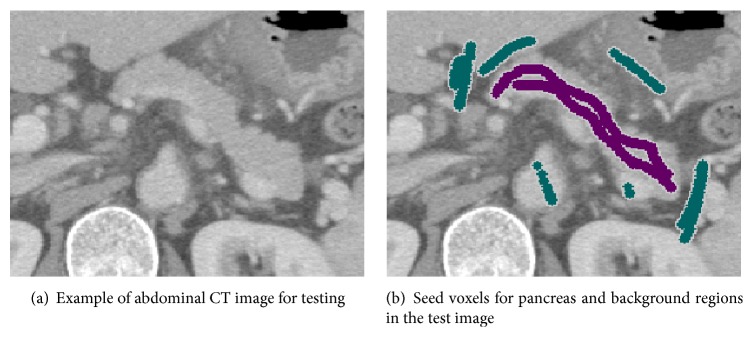
Test CT image and seed voxels.

Figure 3(a) shows an example of an abdominal CT image for testing, and Figure 3(b) shows seed voxels for the test image. The red and blue regions represent seed voxels for pancreas and background regions.

### 2.3. Homography Transformation of Probabilistic Pancreas Atlas

The PA is fitted to the seed voxels through homography transformation which is higher dimensional conversion than simple parallel translation used in our previous paper [12]. Let *A*(*x*, *y*, *z*) denote the probability value of a voxel at (*x*, *y*, *z*) in the PA. The seed images are represented by

(2) $$\begin{array}{c} S\left(\;x,y,z\;\right)=\left\{\begin{array}{cc} -1 & \text{if }\left(x,y,x\right)\in \text{pancreas seeds},\\ \alpha & \text{if }\left(x,y,x\right)\in \text{background seeds},\\ 0 & \text{otherwise}, \end{array}\right. \end{array}$$

where *α* is a coefficient for background.

Homography transformation is defined by

(3) $$\begin{array}{c} P^{\prime }=Hp, \end{array}$$

where **p** = (*x*, *y*, *z*, 1)^*T*^ and **P**′ = (*X*′, *Y*′, *Z*′, *W*′)^*T*^. Homography matrix *H* is represented by

(4) $$\begin{array}{c} H=\left[\begin{array}{cccc} h_{11} & h_{12} & h_{13} & h_{14}\\ h_{21} & h_{22} & h_{23} & h_{24}\\ h_{31} & h_{32} & h_{33} & h_{34}\\ h_{41} & h_{42} & h_{43} & h_{44} \end{array}\right], \end{array}$$

and *W*′ is normalized by

(5) $$\begin{array}{c} P=\frac{1}{W^{\prime }}P^{\prime }, \end{array}$$

where **P** = (*X*, *Y*, *Z*, 1)^*T*^.

By using the Homography transformation *H*, the following energy function,

(6) $$\begin{array}{c} E_{H}\left(\;h_{11},h_{12},\ldots ,h_{44}\;\right)=\underset{x,y,z}{\sum }A\left(\;X,Y,Z\;\right)\cdot S\left(\;x,y,z\;\right), \end{array}$$

is defined to evaluate the fidelity between the PA and the seed voxels. The optimal PA, *A*^*∗*^(*x*, *y*, *z*), is obtained by minimizing the energy function. The steepest descent method [14] is used for minimization in this paper.

**Figure 4:**
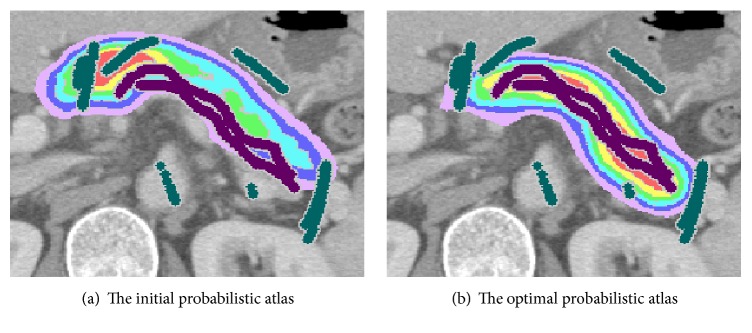
Homography transformation of the probabilistic atlas.

Figure 4 shows a transformation result of the PA whose probabilistic values are represented by color codes. Figures 4(a) and 4(b) show the initial and optimal PAs, respectively. The figure demonstrates that the PA can be well fitted to the pancreas seeds by the transformation introduced here.

### 2.4. Segmentation by Use of Graph Cut Technique

Let *p* ∈ *P* be a voxel in a CT image for testing and *ω*_*p*_ = {PC, BG} ∈ *Ω* be a label assigned to the voxel *p*. PC and BG represent a pancreas and background, respectively. The energy function of the graph cut is defined by

(7) $$\begin{array}{c} E\left(\;\Omega \;\right)=B\left(\;\Omega \;\right)+\lambda \cdot R\left(\;\Omega \;\right). \end{array}$$

*B*(*Ω*) is a smoothing term defined as follows:

(8) $$\begin{array}{c} B\left(\;\Omega \;\right)=\underset{\left\{p,q\right\}\in N}{\sum }B_{\left\{p,q\right\}}\cdot \delta \left(\;\omega_{p},\omega_{q}\;\right), \end{array}$$

where

(9) Bp,q∈N=exp⁡−vp−vq2distp,q,δωp,ωq=1if  ωp≠ωq0otherwise.

*N* represents the neighborhood relationship between voxels and *v*_*p*_ is the value of a voxel *p*. *R*(*Ω*) is a data term defined by

(10) $$\begin{array}{c} R\left(\;\Omega \;\right)=\underset{p\in P}{\sum }R_{p}\left(\;\omega_{p}\;\right), \end{array}$$

and *R*_*p*_(*ω*_*p*_) is defined by

(11) RpPC=−log⁡Pr⁡vp ∣ PC,

(12) RpBG=−log⁡Pr⁡vp ∣ BG.

Pr⁡(*v*_*p*_∣PC) and Pr⁡(*v*_*p*_∣BG) are the conditional probabilities of the voxel values given a pancreas and background, respectively. These conditional probabilities are computed from the histograms of voxel values in pancreas and background seed regions, respectively.

### 2.5. Data Term Weighting with Probabilistic Atlas

The graph cut whose data term is weighted by the optimal PA is applied to the CT image for testing. The PA is used as a priori probability to obtain the a posteriori probabilities of pancreases and background given *v*_*p*_ as follows:

(13) Pr⁡PC ∣ vp∝Pr⁡vp ∣ PC·A∗x,y,z,

(14) Pr⁡BG ∣ vp∝Pr⁡vp ∣ BG·1−A∗x,y,z.

By replacing the conditional probabilities in (11) and (12) with the a posteriori probabilities in (13) and (14), respectively, the PA is used as a weighting factor in the data term of the graph cut.

## 3. Experimental Results

In this study, we used the abdominal CT images of fifteen cases. These CT images were provided by the Japanese Society of Medical Imaging Technology [15]. The image resolutions were 512 × 512 pixels, the pixel sizes were from 0.549~0.625 mm/pixel, and the slice thickness is 2 mm. Pancreas regions were manually extracted from the images and were used as ground truth. We compared not only segmentation accuracy but also operation times and efforts between the conventional graph cut (CGC) method and the atlas-based graph cut (AGC) method proposed in this paper.

### 3.1. Comparison of Segmentation Accuracy

The leave-one-out study was applied to the fifteen CT images. In this study, first, one CT image was chosen as a test image, and the seed voxels for a pancreas and background were interactively selected from the test image by use of the GUI system. A pancreas region was extracted by applying the CGC to the seed voxels in the test image. Segmentation accuracy was evaluated by the following Jaccard index (JI):

(15) $$\begin{array}{c} JI\left(\;E,G\;\right)=\frac{\left|E\cap G\right|}{\left|E\cup G\right|}, \end{array}$$

where *E* is the extracted region and *G* is the ground truth. JI is 1 if *E* and *G* are the same and 0 if they are completely different from each other. Next, from the other (fourteen) CT images, a PA of pancreas regions was constructed, and the AGC was applied to the same seed voxels in the test image. Another JI was calculated in the same manner. These processes were iterated fifteen times by changing the test images.

**Figure 5:**
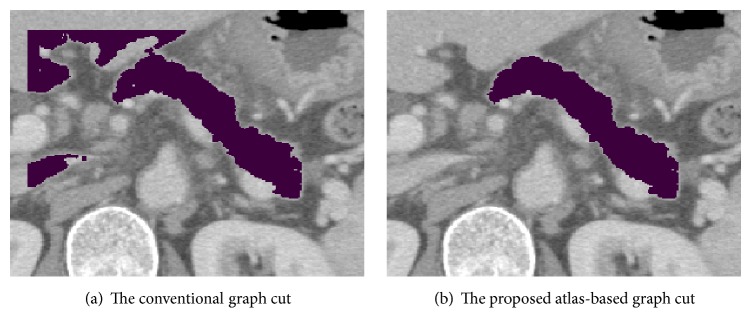
Segmentation results of a pancreas.

Figures 5(a) and 5(b) show the segmentation results of the CGC and the AGC, respectively. The CGC extracted other regions mistakenly, whereas the AGC extracted the pancreas region almost correctly.

Table 1 lists the JIs of the CGC and the AGC. The mean JI of the CGC was 0.325 (*σ* = 0.152), whereas that of the AGC was 0.770 (*σ* = 0.102). The AGC was more accurate than the CGC.

### 3.2. Comparison of Operation Times and Efforts

Another leave-one-out study was applied to eight CT images that were selected for this second experiment. First, the CGC was iteratively applied until the operation time exceeded 600 seconds. Next, the AGC was iteratively applied in the same manner. At each iteration, the GUI system calculated the JIs of extracted regions against the ground truth and recorded operation times and the occurrence numbers of mouse button pressing, mouse wheel scrolling, and mouse dragging.

**Figure 6:**
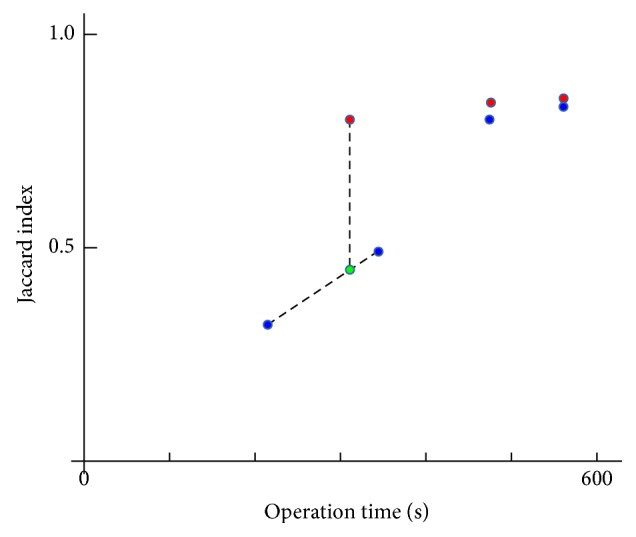
The relation between the Jaccard indices and the operation times.

Figure 6 shows a typical relation between the JIs (the vertical axis) and the operation times (the horizontal axis). The blue and red dots represent the results of the CGC and the AGC, respectively. The green dot is a point interpolated between the first and second segmentation of the CGC. The interpolated point can be considered to correspond to the JI at the first operation time of the AGC. Although the CGC was able to obtain the first segmentation faster than the AGC, the JI of the AGC (the first red dot) was larger than that of the CGC (the green dot).

Table 2 lists the JIs of the AGC at the first operation times and those of the CGC at the corresponding times. The AGC was more accurate than the CGC.

Table 3 shows the relations between user-waiting times (WT) and the occurrence numbers of mouse button pressing (BP), mouse wheel scrolling (WS), and mouse dragging (MD) of the CGC and the AGC. The WT of the AGC is larger than that of the CGC, because the AGC needs to fit the PA to seed voxels. There are no distinct differences in the BP and WS. The MD of the AGC is smaller than that of the CGC.

## 4. Discussion

The main contribution of the proposed method is to combine the anatomical knowledge of medical doctors with the statistical information of pancreas shapes. The anatomical knowledge and statistical information are represented by seed voxels and the PA, respectively. Many segmentation methods used only the statistical information, which was useful to extract the pancreas regions of typical shapes. However, these methods often failed to extract pancreases with irregular shapes that did not appear in training data. The proposed method can deal with such pancreases more flexibly through interaction with medical doctors.

Our previous method [12] only translated the PAs in the testing phase and had difficulty in fitting the PAs to irregular pancreases. The newly proposed method adopted the homography transformation that was higher dimensional conversion. The homography transformation was able to increase the mean JI index from 0.705 in [12] to 0.770 in Table 1.

The proposed method allows medical doctors to modify the segmentation results of the CAD system. There are no 100% segmentation methods, and therefore it is necessary to provide a modification function for medical doctors working in actual medical settings. In the proposed method, they can overwrite new seed voxels onto pancreas regions extracted by the CAD system if necessary.

In general, mouse dragging requires the most efforts among mouse operations, because medical doctors need to move the mouse two-dimensionally on desks. Mouse button pressing and wheel scrolling can be done only by one-dimensional actions. Table 3 demonstrates that the proposed method can reduce the mouse dragging actions and therefore is more effective than the conventional graph cut in this regard. This effectiveness was not shown in our previous paper [12] but becomes evident in the present paper. The proposed method needs more user-waiting time than the conventional graph cut. The atlas fitting and transforming should be more efficient.

The proposed method can be used not only for clinical purposes, but also for research purposes. Many studies related to organ segmentation need ground truth for training and evaluation, but they have not discussed how to produce the ground truth effectively. By use of the proposed method, researchers can obtain the ground truth with less efforts.

Many automatic segmentation methods [16–20] have been proposed, and several methods, such as [21], would outperform our method. We should compare our method with these state-of-the-art methods under equivalent conditions. In addition, the proposed method would require much more manual operations than the automatic methods. It is necessary to evaluate the proposed method considering the tradeoff between these advantages and disadvantages.

The work [22] proposed an organ segmentation method based on PAs and the GC technique. This method used the gradient vectors of signed distances in PAs, whereas our method used the signed distances themselves. These methods should be compared with each other in the future.

## 5. Conclusion

This paper proposed an interactive segmentation method of pancreases in abdominal CT images based on the anatomical knowledge of medical doctors and the statistical information of pancreas shapes. The proposed method was compared with the segmentation method based on the conventional graph cut. The experimental results demonstrated that the proposed method was more accurate and effortless.

## Figures and Tables

**Table 1:** Jaccard indices of the CGC and the AGC.

	CGC	AGC
1	0.158	0.784
2	0.181	0.622
3	0.264	0.808
4	0.238	0.640
5	0.214	0.809
6	0.345	0.825
7	0.370	0.605
8	0.483	0.822
9	0.270	0.741
10	0.728	0.870
11	0.419	0.753
12	0.205	0.632
13	0.482	0.888
14	0.225	0.827
15	0.291	0.927

Mean	0.325	0.770
SD	0.152	0.102

**Table 2:** Jaccard indices of the CGC and the AGC at the same operation times.

	CGC	AGC
1	0.451	0.804
2	0.309	0.620
3	0.416	0.716
4	0.582	0.700
5	0.410	0.700
6	0.597	0.764
7	0.265	0.710
8	0.408	0.841

Mean	0.430	0.732
SD	0.116	0.0691

**Table 3:** Comparison of operation efforts.

	WT		BP		WS		MD	
	CGC	AGC	CGC	AGC	CGC	AGC	CGC	AGC
1	67	125	27	47	658	699	10647	6445
2	71	137	71	72	560	631	11513	7363
3	82	143	23	32	839	832	7297	5135
4	155	162	33	30	709	717	8193	5417
5	132	156	15	30	594	750	11777	7010
6	116	159	19	35	775	633	11187	6875
7	143	150	28	28	647	660	13600	6233
8	113	136	44	53	739	565	11902	6692

Mean	110	146	33	41	690	686	10760	6396
SD	33	12.9	18	15	93.5	82.5	2061	775.2
